# Novel Highly Soluble Chimeric Recombinant Spidroins with High Yield

**DOI:** 10.3390/ijms21186905

**Published:** 2020-09-20

**Authors:** Qiupin Jia, Rui Wen, Qing Meng

**Affiliations:** Institute of Biological Sciences and Biotechnology, Donghua University, Shanghai 201620, China; xibeizi122@126.com (Q.J.); wrhvhgugu@gmail.com (R.W.)

**Keywords:** chimeric recombinant spidroins, aciniform spidroin, hand-drawn fiber, mechanical properties, secondary structure

## Abstract

Spider silk has been a hotspot in the study of biomaterials for more than two decades due to its outstanding mechanical properties. Given that spiders cannot be farmed, and their low silk productivity, many attempts have been made to produce recombinant spidroins as an alternative. Herein, we present novel chimeric recombinant spidroins composed of 1 to 4 repetitive units of aciniform spidroin (AcSp) flanked by the nonrepetitive N- and C-terminal domains of the minor ampullate spidroin (MiSp), all from *Araneus ventricosus.* The spidroins were expressed in the form of inclusion body in *E. coli* with high yield. Remarkably, the aqueous solubility of the four spidroins ranged from 13.4% to over 50% (*m/v*). The four spidroins could self-assemble into silk-like fibers by hand-drawing. The secondary structures of these proteins, determined by circular dichroism spectrum (CD) and Fourier transform infrared spectrum (FTIR), indicated a prominent transformation from *α*-helix to *β*-sheet after fiber formation. The mechanical properties of the hand-drawn fibers showed a positive correlation with the spidroin molecular weight. In summary, this study describes promising biomaterials for further study and wide application.

## 1. Introduction

Spider silks are the toughest known natural silk fibers, and therefore, have received much attention in the field of biomaterials [1,2]. Orb-web spiders have evolved multiple morphologically differentiated silk glands, each of which produce different types of silk fibers with distinct properties for specific tasks [3]. For example, minor ampullate silk produced from the minor ampullate gland displays high tensile strength and is used for the auxiliary spiral of the spider web [4]. Spider silks are mainly composed of spider silk proteins (spidroin) which have extremely high molecular weights, i.e., up to 350 kDa [5], and consist of a common 3-domain-architecture: a nonrepetitive N-terminal domain (NT), an extensive repetitive central domain (Rep), and a nonrepetitive C-terminal domain (CT) [6]. The N- and C-terminal domains are more conserved across different spidroins [7], and both play a critical role in spidroin storage in the gland and the initiation of the fiber assembly process [8], while the central repetitive domain is less conserved and tailored for the individual mechanical functions of the different silk types [9,10]. In general, it consists of modular units with different lengths in different spidroins; the modular units can repeat up to approximately 100 times within the core domain [10,11,12].

Among the seven known types of spider silk, aciniform silk, composed of aciniform spidroin (AcSp) and secreted from aciniform gland, is used as the soft inner silk of the egg sack, and also the silk for swathing prey [3,7,13]. It exhibits excellent extensibility and tensile strength [14], which, together, make it 50% tougher than the strongest known and most studied major ampullate spider silk [15,16]. The exceptional performance is thanks to its distinctive protein structure, especially the repetitive domain [17], which usually has long repetitive units, i.e., mostly longer than 200 amino acids with motifs like (A)_2_, (S)_2-5_ [9] and high Gly, Ala, and Ser contents [7,14], while lacking short repetitive motifs, such as polyGA, GGX, GPGGX, and polyA [18].

In the previous attempts to synthesize new, high performance and sustainable recombinant spidroin materials, researchers produced many recombinant spidroins, including multiple repetitive motifs [19,20] fused with or without nonrepetitive regions [21,22,23,24,25,26,27,28]. However, the yield of these spidroins were usually too low to meet the needs of large scale application, and only rarely could they reach a level of solubility that was comparable to the storage concentration of natural spidroin in the spider gland, most likely resulting in inferior mechanical properties [3]. Therefore, it is necessary to create new recombinant spidroins with high production and excellent solubility to pave the way for the development of high-performance silk fibers.

In this study, we constructed four novel tripartite chimeric spidroins containing the 1 to 4 repetitive units of *Araneus ventricosus* AcSp1 and the N- and C-terminal domains of *Araneus ventricosus* MiSp. By replacing the nonrepetitive domains at both ends of the repetitive unit from AcSp with those from MiSp, we designed a series of spidroins to ensure protein productivity in the prokaryotic expression system, and in the hope of making high-performance silk fibers. The secondary structures of these spidroins were analyzed according to their circular dichroism spectra (CD). All four spidroins could form silk fibers by hand-drawing, which were then characterized by scanning electronic microscopy (SEM), Fourier transform infrared spectroscopy (FTIR), and mechanical property testing.

## 2. Results and Discussions

### 2.1. Spidroin Production and Solubility

**Figure 1:**
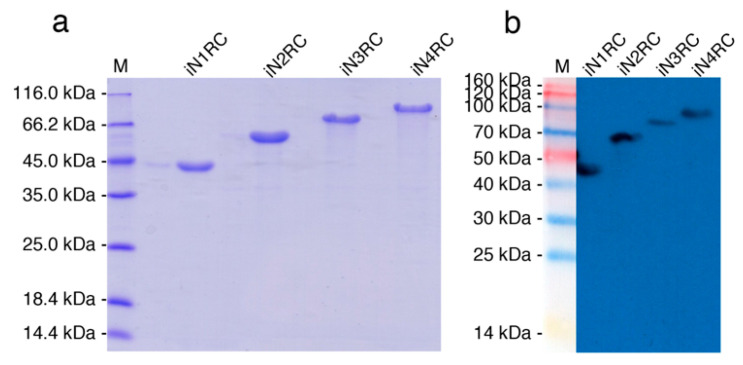
Identification of the recombinant spidroins. Migration of protein size markers are shown in lane M. Purified proteins were analyzed through SDS-PAGE and followed by (**a**) Coomassie brilliant blue staining and (**b**) Western-blot with His-tag antibody.

The sequences of the four constructions were confirmed by both restriction enzyme digestion and sequencing, showing 100% conformity to the anticipated sequences. The four spidroins were successfully expressed as inclusion bodies in *E. coli* cells. Urea denaturation was introduced in the purification procedure, and then urea was removed by dialysis against 20 mM Tris buffer at pH 8.0. The SDS-PAGE analysis (Figure 1a) showed that the purities of the four spidroins were all above 90%, and the result of western-blot (Figure 1b) using anti-His-tag confirmed their identities.

The yield of the iNnRC spidroins was negatively correlated to the protein molecular weight (Table 1), which is in line with previous reports on the productivity of recombinant spidroins [19,21]. The iN1RC spidroin showed the highest yield, i.e., 254 mg per liter shake flask culture, while that of iN4RC was 89 mg/L. By concentrating in 20 mM Tris buffer at pH 8.0, the solubility of the iNnRC spidroins showed a negative correlation to protein size (Table 1), in other words, with increasing repetitive unit R, the solubility of the spidroins gradually declined from more than 500 mg/mL for iN1RC to 134 mg/mL for the largest spidroin, iN4RC. Significantly, the solubility of iN1RC in aqueous buffer (565 mg/mL) was equal to or even higher than the known stored concentration of most natural spidroins and the previously reported recombinant spidroins such as NT2RepCT [22] and NTW_1_CT [21].

**Figure 2:**
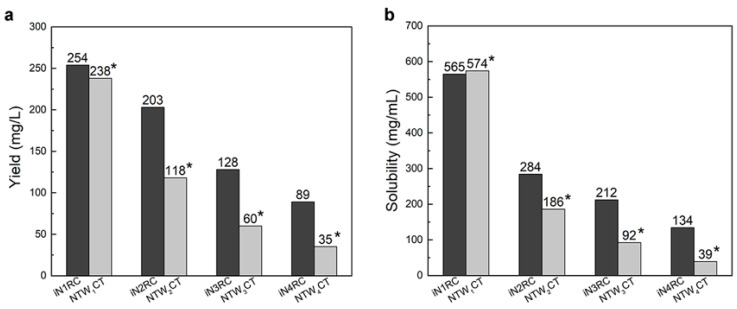
The yield (**a**) and solubility (**b**) of the iNnRC spidroins compared with NTW_1-4_CT spidroins [21]. The yield and solubility of the spidroins are shown above the bars. *Data for NTW_1-4_CT are from [21].

Low yield and low aqueous solubility have been two major obstacles to the biomimetic spinning of recombinant spidroins at a large scale [12,29]. In previous studies, highly-soluble heterologous NT and CT have been employed to address such problems. For example, NT from MaSp of *Euprosthenops australis* and CT from MiSp of *Araneus ventricosus* bracketed repetitive unit(s) of AcSp1 from *Argiope trifasciata* [21] or a short repetitive region from *Euprosthenops australis* [22], resulting in small recombinant spidroins with high yield and solubility; however, when the size of the spidroins was greater than 60 kDa, the yield and solubility both dropped drastically. Herein, we used the same CT as in our previous study [22] and chose an NT with a moderately solubility [22]. It is worth mentioning that although the NT in this study was not as soluble as that in the series of the NTW_1-4_CT spidroins, which are also composed of same-sized 200 amino acid AcSp repetitive units [21,30], and this inferior solubility may have some effect on the protein yield, together with the conditions employed herein and the subsequent purification process, a similar or even higher yield of chimeric recombinant spidroins was obtained, notably of the three larger proteins (a comparison is shown in Figure 2a). Moreover, the yield of iN4RC spidroin was 89 mg/L, the lowest in this study. However, considering that its molecular weight is 104.2 kDa, its yield was higher than that of most reported *E. coli* expressed recombinant spidroins with similar sizes, for example, a previous 94 kDa recombinant flagelliform spidroin Sfl_3_CT [31] only had a yield of 2.5–3 mg/L.

**Figure 3:**
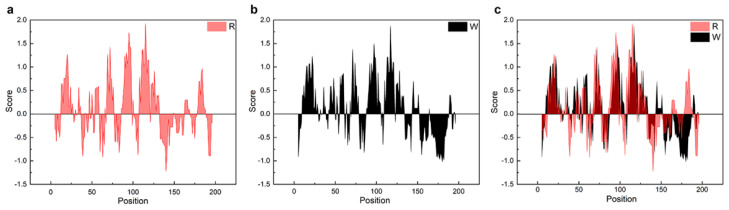
Comparison of the hydropathicity plots of the repetitive units. R, the repetitive unit in iNnRC; W, the repetitive unit in NTW_1-4_CT [21]. (**a**) The hydropathicity plot of R. (**b**) The hydropathicity plot of W. (**c**) The hydropathicity plot shows the overlap between R and W.

Unlike NTW_1-4_CT spidroins [21], iNnRC spidroins were all expressed as inclusion body in *E. coli* cells. This might have been due to the high yield, as overexpression of the recombinant spidroins may result in water-insoluble products in the host cells [12]. Moreover, the iNnRC spidroins in the insoluble fraction may also due to the nature of the NT, as there are two cysteines that could generate disulfide bonds ligating protein monomers and causing aggregation [32]. To isolate and refold the protein aggregate, centrifugation and urea denaturation were used in the purification step. Later, urea was usually removed by gradient-dialysis [21] to ensure biological applicability. However, herein, urea removal was performed by directly dialyzing against 20 mM Tris buffer. The iNnRC spidroins surprisingly remained soluble during dialysis without any obvious precipitation. After concentrating, all four spidroins exhibited high aqueous solubility. This was mostly related to the overall architecture of the spidroins, especially the hydropathicity of the repetitive unit R. In Figure 3, the Kyte and Doolittle hydropathicity of AcSp repetitive unit R used in this study and W used in NTW_1-4_CT [21] were compared. The positive scores indicate hydrophobicity, while the negative scores indicate hydrophily. The curves show that the distribution of hydrophilic and hydrophobic amino acid sequences in R is greater even than in W. The size of R accounts for its increased proportion in the iNnRC spidroins, and the NT and CT are both relatively highly soluble. These features together contribute to the higher solubility of iNnRC spidroins (with 2-4 Rs) compared to NTW_1-4_CT (shown in Figure 2b) and the other reported recombinant spidroins with similar molecular weights [33].

### 2.2. Secondary Structure Analysis of iNnRC Spidroins

**Figure 4:**
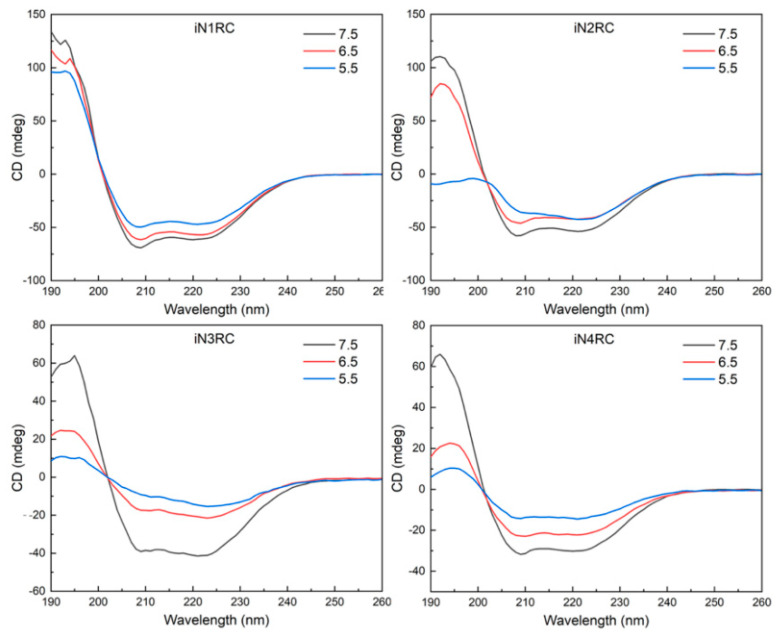
Protein solution stability of the iNnRC spidroins at different pHs. CD spectra of the spidroins from iN1RC to iN4RC were collected at pH 7.5, 6.5, and 5.5 in 20 mM sodium phosphate buffer.

Circular dichroism (CD) spectroscopy and FTIR spectroscopy were performed to investigate the conformational changes in the four recombinant spidroins before and after fiber formation. According to the CD spectroscopy, the four iNnRC spidroins in solution at pH 7.5 all showed spectra curves with positive peaks at 193 nm and two negative peaks at 208 nm and 222 nm (Figure 4). These distinct peaks indicated that the spidroins had an *α*-helix-dominated conformation [34,35]. Despite the different number of repetitive unit R in these four constructs, they all showed similar CD curves, which suggested that the NT and CT, as well as the repetitive unit R, as predicted, were in an *α*-helical conformation [7,30,36]. Additionally, the mainly *α*-helical conformation demonstrated good refold of the iNnRC spidroins after urea denaturation. When the pH decreased from 7.5 through 6.5 to 5.5, the CD spectra of the spidroins showed a decline in the *α*-helix characteristic peaks values for the iN1RC spidroin, while these peaks in spidroins with more than one repetitive R were drastically decreased, resulting in flattened spectra curves and indicating a distinct loss of *α*-helical structure.

**Figure 5:**
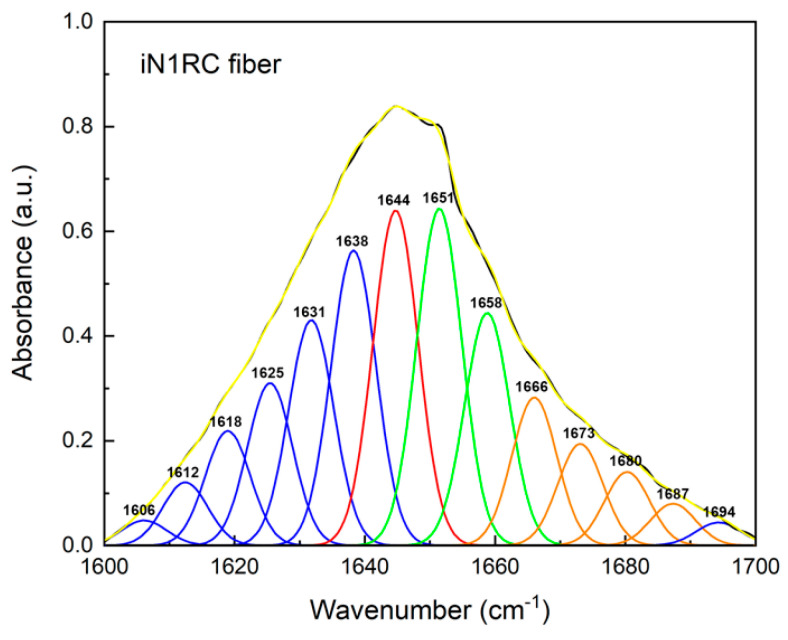
FTIR spectra of iN1RC fiber. The decomposition of the iN1RC fiber spectrum in the amide I region (1600~1700 cm^−1^), and the peaks in blue, red, green, and orange correspond to the secondary structures of *β*-sheet, random coil, *α*-helix, and *β*-turn, respectively, while the black curve corresponds to the original curve and the yellow curve corresponds to the sum of the decomposed spectra.

FTIR spectroscopy was used to investigate the secondary structure contents of the spidroin fibers. All four spidroin fibers showed similar spectra, regardless of the different quantities of repetitive unit R. A typical decomposition of the amide I band (1600~1700 cm^−1^) in the iN1RC fiber is shown in Figure 5, and the calculated secondary structure contents of the iNnRC fibers are shown in Table 2. FTIR of the iNnRC fibers indicated a primary *β*-sheet conformation of 41–44%, while *α*-helix was around 26%. Compared with the major secondary structure content of the spidroins in solution, there was a distinct conformational conversion from *α*-helix to *β*-sheet, which also occurs in natural spider silk formation [9]. When compared with the NTW_1-4_CT spidroins [21], the iNnRC fibers exhibited more *β*-sheets. It is assumed that the *β*-sheet is responsible for the fiber’s strength [37], and this most likely lays the foundation for the superior mechanical properties of iNnRC fibers.

### 2.3. The Morphology and Mechanical Properties of the iNnRC Fibers

**Figure 6:**
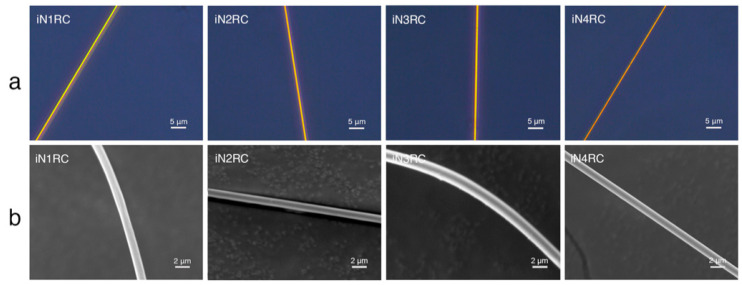
Morphological characterization of the iNnRC fibers. (**a**) Inverted phase contrast microscope images and (**b**) scanning electron microscope images.

All iNnRC spidroins could form silk-like fibers longer than 10 cm by hand-drawing from Tris buffer. Inverted phase-contrast microscopy and scanning electron microscopy were used to study the morphology of the fibers. As shown in Figure 6 and Table 3, all the fibers from iN1RC to iN4RC had a smooth surface and a diameter of 1.8 μm, except iN3RC, which had a diameter of 2.5 μm. The fibers presented here were similar in appearance of native spider silk, but larger in diameter compared to natural aciniform silk [15].

**Figure 7:**
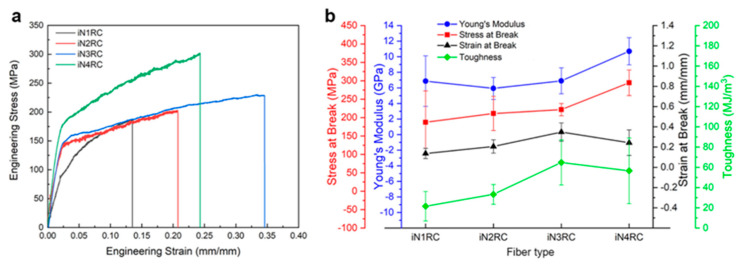
Mechanical properties of iNnRC fibers. (**a**) Stress-strain curves of representative iNnRC fibers. (**b**) Summary of the mechanical properties of the fibers.

The mechanical properties of the fibers were determined using fiber samples with a length of 10 mm with a smooth surface and even thickness. Typical stress−strain curves and a summary of the iNnRC spidroin fibers are shown in Figure 7 and Table 3 respectively, where “stress” defines the tensile strength and “strain” the extensibility [38]. The curves in Figure 7a show the two typical stages of the silk fiber, which indicates that the fibers had qualitatively similar stress–strain properties to native spider silk, in that they displayed an initial elastic phase up until a yielding point, after which plastic deformation occurred [22]. In Figure 7b, the tensile strength of the iNnRC fibers showed a positive correlation with increasing repetitive units in the spidroins. However, the toughness did not show such a tendency from iN3RC to iN4RC, and this break was most likely due to the thicker fiber samples tested for the iN3RC. According to a previous reported, the thickness of the regenerated silk fiber is highly correlated to its tensile strength and strain, which furthermore affects fiber toughness [39]. In this study, the average diameter of the iN3RC fibers was 2.5 μm, while those of the other three types of fibers were 1.6 ~ 1.8 μm (shown in Table 3). The larger diameter of iN3RC led to better extensibility and toughness (34%, 64.8 MJ/m^3^), i.e., even better than the iN4RC fibers (24%, 56.5 MJ/m^3^). But when we removed the iN3RC fibers, and compared the remaining fibers with similar average diameters, the mechanical properties demonstrated a positive correlation with increasing repetitive units R in each spidroin. Notably, with approximately one-third the molecular weight of natural AcSp spidroin [15], the tensile strength of the iN4RC fiber (295 ± 35 MPa) was almost half that of its natural counterpart (687 ± 56 MPa) [15], while with a molecular weight of less than 1/3 of that of natural AcSp spidroin, the extensibility of the iN3RC fiber (34 ± 0.09%) was ten percent shy of half of the natural aciniform silk extensibility (86 ± 0.03%) [15].

Compared with the mechanical properties of NTW_1-4_CT fibers [21], the extensibility of the fibers was quite similar; however, there was a great difference in the tensile strength of the fibers. All the iNnRC fibers were stronger than their correspondent NTW_1-4_CT fibers, especially those with 1 to 2 repetitive units. As mentioned above, there were two cysteines in the NT of the iNnRC spidroins which could form disulfide bonds during fiber formation, giving the fiber more solid inter- and/or intra-molecular covalent bond crosslinking to stabilize the protein structure and improve its tensile strength. Since the repetitive unit R and W were derived from different spider species, their amino acid compositions shared some degree of similarity, but still with diversity, which might have played a big role in the observed differences in mechanical properties. Further experiments are needed to investigate this in detail.

## 3. Materials and Methods

### 3.1. Construction of Recombinant Spidroins

**Figure 8:**
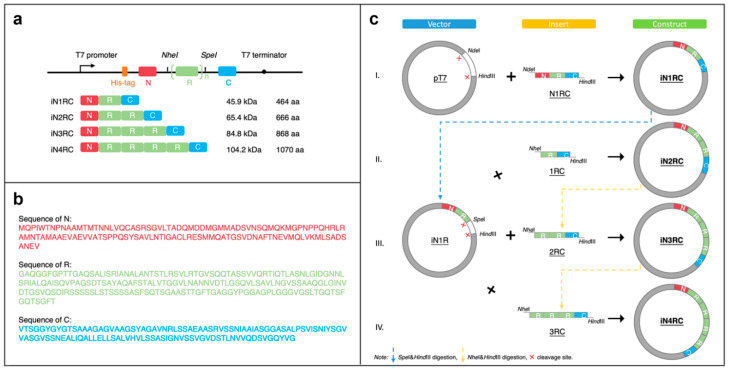
Construction of the iNnRC spidroins. (**a**) Schematic illustration of the iN1RC to iN4RC spidroins with their sizes (A His-tag was added to the N-terminal domain of all the four recombinant spidroin constructs). (**b**) Amino acid sequences of the three fragments. (**c**) Schematic illustration of the construction strategy of the recombinant genes. The plasmids of iN1RC to iN4RC were constructed through Step I/II/III/IV.

The sequence of a repetitive unit (R) was from *Araneus ventricosus* AcSp1 (GeneBank accession number MG021196), and the N- and C- terminal (N and C) sequences were from *Araneus ventricosus* MiSp (GeneBank accession number JX513956) (Figure 8b). Four recombinant plasmids were constructed with the iNnRC (n = 1, 2, 3, and 4) sequences containing 1 to 4 repetitive units, as well as nonrepetitive terminal domains on each side (Figure 8a).

Primer R1 and R2 (Table 4) were used to amplify the fragment R and introduce the restriction enzyme sites *Kpn*I-*Nhe*I and *Spe*I-*EcoR*I on each side. Primer N1 and N2 (Table 1) were used for the amplification of the fragment N, introducing *Nde*I and *Kpn*I-*Hind*III restriction sites at each end. Primer C1 and C2 (Table 4) were for fragment C, and ended up with *EcoR*I and *Hind*III restriction sites on each side. To obtain the iN1RC construct, the three fragments were digested with the restriction enzymes mentioned above, and then ligated into a pT7 plasmid.

The constructs of iN2RC to iN4RC were constructed according to the previously reported “head-to-tail” cloning strategy [19,40,41]. In brief, the iN1RC plasmid was digested with restriction enzymes *Spe*I and *Hind*III, and the resulting vector fragment containing the N1R coding sequence was ligated with a *Nhe*I-*Hind*III fragment isolated from iN1RC plasmid containing the 1RC coding sequence to obtain the iN2RC plasmid (Figure 8c). By repeating this procedure, iN3RC and iN4RC were produced.

All four constructs were sequenced to ensure all the inserted gene sequences were correct.

### 3.2. Expression and Purification of the Recombinant Proteins

The four constructed plasmids were transformed into *E. coli* expression strain BL21 (DE3) (Tiangen, China). To express the desired protein, the respective iNnRC containing *E. coli* cells was cultured overnight in LB media with 70 mg/L kanamycin at 37 °C and 180 rpm. Then, 10 mL of the overnight culture was inoculated to a 1 L LB media with the same amount of kanamycin and cultured at 30 °C and 180 rpm to an OD_600_ of 0.8~1.0. Isopropylthiogalactoside (IPTG) was added to a final concentration of 0.3 mM to induce protein expression. Then, the cells were cultured overnight at 20 °C and 220 rpm. The cells were harvested through centrifugation (4500 rpm, 20 min, 4 °C), resuspended in 30 mL 20 mM Tris buffer at pH 8.0, frozen at –20 °C for at least 12 h, and then lysed using a Pressure Cell Press JN-3000 Plus (JNBIO, China).

The cell lysate was centrifuged at 4500 rpm and 4 °C for 20 min, and most of the four proteins of interest were found in the insoluble fraction. The pellets were collected and resuspended in 30 mL 20 mM Tris buffer at pH 8.0 with 1 M urea (for iN1RC) or 2 M urea (for iN2RC, iN3RC, and iN4RC), and incubated in an ice-water bath for 3 h on a horizontal rotator (Nuomi, Taizhou, China) at 50 rpm, before being centrifuged at 4500 rpm and 4 °C for 20 min to remove the supernatant. The pellets were then resuspended again in 30 mL 20 mM Tris buffer at pH 8.0 with 6 M urea, incubated at low temperature (as above) for 1 h and centrifuged at 4500 rpm and 4 °C for 20 min to collect the supernatant. The supernatant containing the solubilized target protein was dialyzed against 20 mM Tris buffer at pH 8.0 and 4 °C in a large volume several times to remove urea. After dialysis, the protein solution was centrifuged at 12,000 rpm and 4 °C for 20 min to obtain a soluble protein without any precipitation. Absorbance at 280 nm was measured to determine the protein yield and purified protein concentration. SDS-PAGE and Western-blot (His-tag antibody) were used to check the protein purity and identify the expressed protein. The protein purity was measured using the SDS-PAGE gel in the ImageJ software.

### 3.3. Recombinant Protein Solubility Determination

The four purified and dialyzed proteins were concentrated using Amicon Ultra-15 Centrifugal filter tubes (Millipore, USA) at 3500× *g*, 15 min/per round, and 4 °C until visible precipitates appeared. Then, the concentrated protein was centrifugated at 12,000× *g* and 4 °C for 30 min to remove precipitates, and the protein solubility, e.g., the concentration of the protein, was measured by checking the absorbance of a diluted protein sample at 280 nm (diluting ratio 1:200).

### 3.4. Circular Dichroism (CD) Spectrum Analysis

Recombinant spidroins were analyzed using a chirascan spectrometer (Applied Photophysics Ltd., UK) to collect CD spectra from 260 to 190 nm at 25 °C in a 1-mm path length quartz cuvette. The wavelength step was 1 nm, response time 1 s, and bandwidth 1 nm. The protein samples were prepared by firstly dialyzing into 20 mM sodium phosphate buffer at pH 8.0, and then diluting in 20 mM sodium phosphate buffer at pH 7.5, 6.5, and 5.5 to a final concentration of 0.3–0.6 mg/mL. Each protein sample was scanned three times and the spectra were averaged.

### 3.5. Fiber Hand-Drawing Procedures

Fibers were produced using similar methods as described before [21,28]. Fibers were pulled from four purified protein solutions in 20 mM Tris buffer at pH 8.0 and room temperature. Normally, a 20 μL protein solution (~1 mg/mL) was placed on a glass slide, and silk-like fibers were pulled from the solution using small tweezers or a plastic pipette tip at a continuous pulling speed of ~5 mm/s.

### 3.6. Scanning Electron Microscopy (SEM)

Dry fibers were placed on a scanning electron microscopy (Phenom-World BV, The Netherlands) stub, coated with gold for 45 s, loaded on the microscope stage, observed, and photographed at an acceleration voltage of 10 kV and room temperature.

### 3.7. Mechanical Tests of Silk Fibers

Inverted phase-contrast microscopy was performed using a Leica DMi8 (Leica Microsystems, Wetzlar, German) to photograph fibers at 400× magnification. For each fiber, three locations along the fiber in each photograph were chosen to calculate the average value of the fiber diameter with the ImageJ software.

The mechanical properties of fibers with even thicknesses and without knots were tested on an Agilent T150 UTM instrument with nanomechanical actuating transducer (Agilent Technologies Inc, Santa Clara, CA, USA) at 20–25 °C and ~50% humidity. These fibers were stretched at a constant rate of 0.2% strain/s until the fibers broke. The average mechanical properties of each type of iNnRC fiber were determined from at least 8 samples. Agilent NanoSuite software was used to calculate engineering stress and strain at break, Young’s modulus, and toughness.

### 3.8. Fourier Transform Infrared (FTIR) Spectroscopy

FTIR measurement of silk fibers was performed on a Nicoletln10MX FTIR instrument (Thermo Fisher Scientific, Waltham, MA, USA) between 4000 and 600 cm^−1^ at room temperature. For each spectrum, 64 scans were collected with a resolution of 4 cm^−1^ and recorded through OMNC v8.2 (Thermo Fisher Scientific, Waltham, MA, USA). Three spectra were obtained and averaged for each type of silk fiber, and the blank background spectrum was subtracted before analysis. The spectra were curve-fitted with Gaussian peak shapes. Half-bandwidth was set to 7.42 cm^−1^ and the noise target was set to 1. The spectra analysis was conducted with PeakFit software v4.12 (SPSS Inc. Chicago, IL, USA) and a linear baseline was subtracted from the amide I band (1600–1700 cm^−1^) before curve fitting. Secondary structure contents were estimated by calculating the band range proportion correlated to each secondary structure type, e.g., *α*-helix, *β*-sheet, random coil, and *β*-turn [42,43,44].

### 3.9. Sequence Analysis

The ExPASy tools (SIB, Lausanne, Switzerland, http://www.expasy.org/) were applied for the analysis of the protein sequences and Kyte and Doolittle hydropathicity predictions. 

## 4. Conclusion

In this study, we produced a series of novel recombinant spidroins based on 1 to 4 repetitive units of AcSp, NT, and CT of MiSp from *Araneus ventricosus.* The four spidroins were all produced as inclusion bodies in low-cost *E. coli* cells with high yield, which could be easily isolated by centrifugation and purified through urea solubilization. It is noteworthy that after urea removal by direct dialysis, all spidroins showed high aqueous solubility, with iN1RC being over 50% (*m/v*), which is comparable to natural or recombinant spidroins. Hand-drawn iNnRC fibers exhibited better mechanical properties than previously reported fibers produced from recombinant spidroins with similar sizes and compositions. Notably, the tensile strength of the iN4RC fibers was close to half that of natural aciniform spider silk. Our highly soluble chimeric recombinant spidroins combining easy purification and high yield provide high-performance artificial fibers for future biomaterial applications.

## Figures and Tables

**Table 1:** Protein sizes, Average yield, and Solubility of the iNnRC spidroins.

	iN1RC	iN2RC	iN3RC	iN4RC
Protein size (kDa)	45.9	65.4	84.8	104.2
yield (mg/L)	254	203	128	89
Solubility (mg/mL)	565	284	212	134

**Table 2:** Secondary structure contents of the iNnRC fibers.

	iN1RC (%)	iN2RC (%)	iN3RC (%)	iN4RC (%)
*α*-helix	26.2	27.6	25.2	27.8
*β*-sheet	41.7	41.7	44.2	41.3
*β*-turn	16.8	15.8	15.9	16.7
Random coil	15.3	14.9	14.7	14.2

**Table 3:** Mechanical Properties of the iNnRC Fibers.

Fiber Type	Diameter	Young’s Modulus	Stress at Break	Strain at Break	Toughness
(number)	(μm)	(GPa)	(MPa)	(mm/mm)	(MJ/m^3^)
iN1RC (10)	1.6 ± 0.5	6.9 ± 3.2	187 ± 85	0.13 ± 0.05	21.5 ± 14.6
iN2RC (9)	1.8 ± 0.3	5.9 ± 1.4	211 ± 47	0.21 ± 0.06	33.3 ± 9.8
iN3RC (8)	2.5 ± 0.6	6.9 ± 1.7	221 ± 17	0.34 ± 0.09	64.8 ± 22.2
iN4RC (9)	1.8 ± 0.3	10.7 ± 1.7	295 ± 35	0.24 ± 0.12	56.5 ± 32.5
Native silk *	0.35 ± 0.01	9.8 ± 3.8	687 ± 56	0.86 ± 0.03	379 ± 39

*Aciniform silk from Argiope trifasciata. and data from ref 15. Mean value ± standard error.

**Table 4:** The primer sequences.

Primers	Primer Sequences (5′-3′)
N1	aatgCATATGcaaccaatctggaccaacccaaatg
N2	gatgAAGCTTGGTACCtacttcattcgcgctatccgcagataac
R1	aatgGGTACCGCTAGCggcgcccaaggaggtttcgg
R2	aatgGAATTCccACTAGTagtaaagcctgatgtttgaccgaaag
C1	ctggGAATTCggttacatctggag
C2	cgagAAGCTTtcattaacctacatattggc

Note: the capitalized sequences indicated the restriction enzyme recognition sites.
